# The combination therapy of high-intensity focused ultrasound with radiotherapy in locally advanced pancreatic carcinoma

**DOI:** 10.1186/s12957-016-0809-5

**Published:** 2016-02-29

**Authors:** Yu-Jiang Li, Gui-Lin Huang, Xu-Ling Sun, Xin-Chun Zhao, Zhi-Gang Li

**Affiliations:** Second Department of General Surgery, the First Affiliated Hospital of Medical College, Shihezi University, Xinjiang Shihezi City, 832008 China

**Keywords:** High-intensity focused ultrasound, Radiotherapy, Locally advanced pancreatic carcinoma

## Abstract

**Background:**

The aim of the present study is to evaluate the effectiveness of the combined application of high-intensity focused ultrasound (HIFU) and radiotherapy in the treatment of locally advanced pancreatic carcinoma (LAPC).

**Methods:**

A total number of sixteen patients with LAPC started treatment beginning with HIFU and radiotherapy 1 week after the HIFU treatment. Evaluation of the effectiveness of treatment was performed using main clinical symptoms, serum levels of CA-19-9, Response Evaluation Criteria in Solid Tumors (RECIST) guidelines, and the Kaplan-Meier method for estimating median overall survival (OS). The occurrence of adverse reactions was recorded.

**Results:**

The main clinical symptoms including abdominal pain and lower back pain were alleviated, and the mean visual analog scale (VAS) pain score declined from 5.1 points to just 3.3 points immediately after the HIFU treatment. The median pain relief time was 5.6 months after radiotherapy, serum CA-19-9 levels began to decrease significantly 1 week after the HIFU treatment, from 102.1 to 60.8 U/ml, and the median continuous decline time was 4.3 months after radiotherapy. Partial response (PR) was observed in seven of sixteen patients, with stable disease (SD) in four patients, and progressive disease (PD) in the remaining five patients at 6 months after radiotherapy. Serum levels of amylopsin and lipase were not elevated to abnormal levels. The median OS was 14 months. No serious adverse reactions occurred.

**Conclusions:**

Treatment with both HIFU and radiotherapy can quickly improve symptoms and the quality of life and prolong survival lengths. This combination might be a promising therapeutic treatment for patients with LAPC.

## Background

According to the National Comprehensive Cancer Network (NCCN) guidelines, locally advanced pancreatic carcinoma (LAPC) can be defined as unresectable tumors involving the celiac axis and the superior mesenteric artery but that do not include distant metastasis. LAPC patients make up 25 to 30 % of all pancreatic cancer cases at the time of diagnosis and are not suitable for surgical resection [1, 2]. The median survival time for LAPC is 6 to 10 months [3, 4]. Serious abdominal pain is the most common presentation of this disease, which severely affects the quality of life and the survival periods of the patients. Currently, chemotherapy and radiotherapy are the two main treatment options for LAPC. Although chemotherapy using gemcitabine (GEM) has recently become the standard systemic therapy, this treatment is insufficient for LAPC because the clinical benefit response of chemotherapy is only 23.8 % [5]. Furthermore, a study has suggested that chemotherapy is not very effective for pain relief and causes serious adverse effects [6]. The use of radiotherapy for LAPC is limited because peripancreatic organs such as the duodenum, liver, stomach, and spinal cord have poor tolerance for radiation, and, as a result, adequate radiation doses cannot be delivered to the pancreatic carcinoma. Therefore, a study proposes that if radiation therapy is to be used for patients with LAPC, it is not recommended to be used alone [7].

High-intensity focused ultrasound (HIFU) therapy can ablate various solid tumors with thermal effects produced by ultrasound waves in a noninvasive manner. Several studies have shown that HIFU can successfully and safely ablate LAPC with over 80 % of the patients achieving pain relief, and the overall survival of patients can reach as long as 12.4 months [8–10]. HIFU can ablate the internal structure of tumors and remove the bulk of the tumor burden but can potentially leave behind a small residue of the periphery of tumors. This may lead to recurrence or metastasis, which can be effectively treated with radiation. Therefore, it is worth studying if a combination of HIFU and radiotherapy can provide an optimal therapeutic effect. The purpose of this study was to share our experience in treating LAPC with a combination of HIFU and radiotherapy.

## Methods

### Patients

A total number of 16 patients with LAPC were prospectively enrolled from January 2013 to March 2014, and the study protocol was approved by the Ethics Committee of First Affiliated Hospital of Medical College of Shihezi University. All patients were informed of the potential benefits and risks of the therapy, and written informed consents were obtained.

The inclusion criteria were as follows: (1) LAPC was confirmed pathologically through pancreatic biopsies and computed tomography (CT) images or magnetic resonance imaging (MRI), and no tumor metastasis was found; (2) a single tumor located in the head or body of the pancreas; (3) Karnofsky Performance Status Scale (KPS) of patients was above 80 points; (4) the patients had received no prior treatments including chemotherapy or other invasive treatments; (5) the patients experienced chronic upper abdominal and upper back pain, and the visual analog scale (VAS) was over 3 points; (6) the lesions were more apparent through ultrasound imaging and did not affect the surrounding organs at the preoperative location; and (7) the patients had no uncontrolled, serious diseases such as diabetes, hypertension, or heart disease.

### Treatment

#### HIFU treatment

All patients were examined using enhanced CT or MRI imaging before and after HIFU treatment. All patients received intestinal cleaning with polyethylene glycol electrolyte powder and cleansing enema and skin preparation including degreasing before the treatment. During the procedures, the patients were given an intravenous sedative (midazolam, 3 mg) and an analgesic (fentanyl, 200 μg) to relieve discomfort and to prevent voluntary or involuntary bowel movements.

The patients were placed face down on a water sac filled with degassed water on the treatment table with transducers located at the bottom of the sac. The epigastrium was submerged in the degassed water, and a water balloon was placed between the abdominal wall and the transducers to push the stomach and the intestines into the HIFU beam path. The organs surrounding the lesion were displayed using real-time ultrasonographic imaging. The HIFU treatment began from the deep end to the shallow end of the thickest part of the tumor, slice by slice using a total focal range of 5 to 10 mm. The thickness of the sections was set to 5 mm, and the focus was at least 1.0 cm away from the boundary of the tumor to prevent damage to the surrounding normal tissue. The treatment frequency was 0.8 MHz, the treatment power was 300 W, and the radiation time of each treatment point was 20 to 30 s. After treatment, a 24-h fast was recommended to all patients, in order to protect the surrounding stomach and intestines.

#### Radiotherapy treatment

Three-dimensional conformal radiotherapy was scheduled for 1 week after HIFU treatment. An initial localization was performed. The patient was placed in the supine position and immobilized to the platform using a body mold. Reference points were marked on the body mold, and plain and enhanced CT scans (slice thickness of 2.5 mm) were obtained from the location of interest. The CT images were transferred to a workstation, and treatment plans were developed using a treatment planning system. A medical linear accelerator (2100C/D) was purchased from Varian Inc. (USA). Radiotherapy was delivered using 6 MV photons at 1.5 Gy per fraction, once a day for 5 days a week, for a total dose of 45 Gy, with a required field reduction after 33 Gy.

#### Patient evaluation

VAS pain scores were recorded immediately after HIFU treatment and every week thereafter until the pain progression, which was defined as the pain intensity or analgesic requirements, returned to pre-treatment levels. Amylopsin and lipase levels were measured immediately and 1 week after HIFU treatment. Serum CA-19-9 levels were measured 1 week after HIFU treatment and every month thereafter until rise.

According to the Response Evaluation Criteria in Solid Tumors (RECIST) guidelines [11], complete response (CR) was defined as the absence of all target lesions, a partial response (PR) and progressive disease (PD) as a greater than 30 % decrease and a greater than 20 % increase in the sum of the longest diameters of the target lesions, respectively, and stable disease (SD) as neither PR nor PD.

Pain relief time was defined as the time from the date of alleviating pain after the completion of the treatment to the first documentation of progressive pain or death. Overall survival (OS) time was defined as the time from the initial treatment to the death from any cause or the date of the last follow-up.

Related adverse events such as burns, abdominal pain, pancreatitis, jaundice, hemorrhage, gastrointestinal perforation, and intestinal necrosis were documented. The degree of severity of adverse effects is based on the unified standardized Society of Interventional Radiology (SIR) grading system [12].

#### Statistical analysis

All data were analyzed by using SPSS19.0 software (SPSS, IBM Company, USA). The data are presented as the mean ± SD for normally distributed data or medians for non-normally distributed data. Independent sample *t* tests and Mann-Whitney *U* tests were used for the analysis of normally distributed data and non-normally distributed data, respectively. Survival curves were calculated using the Kaplan-Meier method.

## Results

### Patients and tumors

The procedures were successfully performed on all patients. The average age of the patients was 62.3 years old, and 62.5 % of them were male. Their KPS averaged 84.4 points on a 100-point scale, and the average VAS pain score was 5.1 points. A majority of the tumors were located at the head of the pancreas, while the others were located in the body of the pancreas, and the mean maximum diameter was 3.7 cm (Table 1).

**Table 1:** Patient and tumor characteristics

Characteristics	Value (%)
Patient number	16
Sex	
Male	10 (62.5)
Female	6 (37.5)
Age (year)	62.3 ± 10.5 (49–72)
Karnofsky performance status	84.4 ± 5.1 (80–90)
BMI^a^ (kg/m^2^)	23.8 ± 3.1 (17.3–27.2)
Tumor location	
Head	9 (56.3)
Body	7 (43.7)
Tumor diameter (cm)	3.7 ± 2.6 (3.0–6.2)
Serum CA-199 pre-HIFU (U/L)	102.1 (25.8–229.6)
Serum amylopsin and lipase pre-HIFU (U/L)	101.5 (49.3–202.6)/210.5 (73.7–401.1)
Visual analog scale(VAS)	5.1 ± 2.2 (3–8)

^a^ *BMI* Body mass index

### The effectiveness of treatment

**Fig 1:**
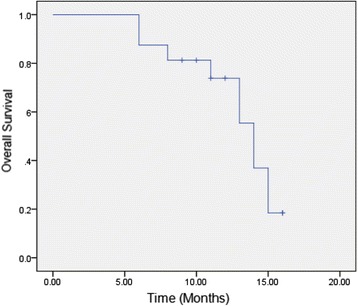
The Kaplan-Meier survival curve of all 16 patients. The median overall survival (OS) time was 14 months (95 % CI 8.0–15.5)

The effectiveness of treatment for all patients was evaluated. The follow-up period was 6–17 months after treatment. Three cases were lost to follow-up at 10, 11, and 13 months, and the remaining cases died within the follow-up period. The mean ablation rate was 71.3 %(50.3–92.1 %) immediately after HIFU treatment, which indicated tumor tissue necrosis, the rate of which was measured using enhanced MRI, CT, or ultrasound. VAS pain score declined from 5.1 points to just 3.3 points immediately after HIFU treatment. The median duration of pain relief time was 5.6 months after the radiotherapy. Pain was improved in the majority of patients; the total effective rate was 93.8 % (15/16). Serum CA-19-9 levels began to fall significantly a week after HIFU treatment, from 102.1 to 60.8 U/ml, and the median continuous decline time was 4.3 months after radiotherapy (Table 2). PR, SD, and PD rates were 43.5, 25, and 31.3 %, respectively, at 6 months after radiotherapy (Table 2). OS is shown in Fig. 1, and the median survival time of patients was 14.0 months.

**Table 2:** Treatment results of tumor

Variable	Value
Pain relief time (month)	5.6 (3.4–8.5)
Serum CA-199 continuous decline time (month)	4.3 (3.0–8.1)
Serum amylopsin and lipase post-HIFU (U/L)	115.8 (56.7–210.2)/222.2 (76.9–417.4)
RECIST(6 months after treatment)	
PR (%)	43.7
SD (%)	25.0
PD (%)	31.3
The median overall survival (OS) time (month)	14 (8.0–15.5)
Incidences of adverse event (SIRC) (%)	12.5 (2/16)

### Adverse effects

There were no differences in the serum levels of amylopsin and lipase before and after the HIFU treatment (Tables 1 and 2), and none of the patients developed clinical pancreatitis. HIFU treatment-related adverse reactions such as burns, bleeding, infections, gastrointestinal tract perforation, and tumor hemorrhage were not observed in all patients. During radiotherapy and within the first 4 weeks after radiotherapy, three patients (18.8 %) experienced grade A–B adverse events including fatigue, nausea, and abdominal distension, which were treated without hospitalization. Two patients (12.5 %) experienced gastrointestinal ulcers, a grade C adverse event, and needed hospital care (Table 2). There were no other serious adverse events related to HIFU treatment and radiotherapy during the follow-up period.

## Discussion

Approximately 73 % of patients with pancreatic carcinoma presented serious pain [13], and it severely affected the quality of life and the survival rate of the patients; previous studies [14] had shown that pain was an independent prognostic factor for the overall survival. Current therapeutic methods such as chemotherapy and radiotherapy are usually offered to patients and are often ineffective at relieving pain. Research shows that the median survival time could be as long as 11 months after radiotherapy alone [15], but remission rates of pain induced by pancreatic carcinoma are only approximately 29 % with radiotherapy monotherapy [6]. Moreover, severe and acute radiotherapy-related toxicities are relatively common, including severe enteritis, ulceration, and perforation of gastrointestinal tract [16]. In some recent years, other techniques such as radiofrequency, cryoablation, microwave, and electroporation had been applied to the treatment of LAPC. Despite a 50–100 % remission rates of pain, but as most of treatment methods were performed during operation, the mortality and other adverse reactions increased significantly [17]. HIFU is a new, noninvasive technology and holds promise in the field of pancreatic cancer treatment. Numerous studies have confirmed the safety and effectiveness of HIFU treatment for LAPC [10, 18, 19], especially in providing pain relief after treatment. Along with its unique advantages, the total remission rate of pain with HIFU treatment was 87.5 to 100 % [8, 20]. In addition, HIFU as a thermotherapy might be an effective way of augmenting radiation therapy [21]. Given the above data, the combination therapy of HIFU with radiotherapy for LAPC might have good therapeutic effects in clinical practice.

In our subjects, the combined application of HIFU and radiotherapy significantly relieved pain in most patients and had longer remission durations compared to simple HIFU, which only resulted in a 10-week remission [20]. The causes for pain relief after HIFU treatment are not fully understood, but researchers have proposed the following mechanisms [6, 22, 23]: (1) the thermal effects of HIFU might damage the nerve fibers which were infiltrated by the tumor; (2) the solar plexus in the target area might be damaged by a long ultrasonic radiation in the far field and caused the pain signal to the brain to be blocked; (3) the pressure within the tumor decreased after treatment causing the pressure on the nerve to be reduced; and (4) HIFU may also result in acoustic pressure and cavitation-driven changes in ion flux through both existing ion channels and defects formed in the neuronal membrane. The experimental results proved that HIFU can induce transient neuromodulation and resulted in changes to the neuronal excitation levels. Moreover, radiotherapy provided a good local control for LAPC and helped patients have a longer pain-free period. In this study, one patient’s pain was not alleviated after HIFU treatment, and we suspect that it might be related to a low ablation rate. Pain control is an important aspect of LAPC treatment; it can significantly improve quality of life and prolong the patients’ lifespan. This study confirms both of these aspects regarding HIFU treatment.

HIFU can prolong the survival period of the patient. Previous research has shown that, with HIFU treatment, 17.5 % of cases of primary tumors experienced PR, and 70 % of the cases experienced SD, and the overall median survival periods were 10 and 12.4 months, respectively [10, 20]. Thus, HIFU has an advantage over either radiotherapy or chemotherapy alone [15, 24]. In our study, the PR and SD were 43.5 and 25 %, respectively, and the overall median survival period was 14 months. The combination of HIFU and radiotherapy has obvious advantages, including simple treatment, higher PR rate, and prolongation of survival, which are similar to chemoradiation [25, 26]. The levels of tumor marker CA-19-9 decreased in patients after treatment and remained at lower levels for a long time, which indicates that LAPC was controlled well.

In terms of the adverse reactions, the combined therapy decreased the dose of external irradiation, so the radiation-related complications were reduced and comparable to those of simple radiotherapy. No serious adverse reactions occurred during or after the combined application of HIFU and radiotherapy in this study. Compared with chemoradiation, toxicity and the treatment cycle were greatly reduced [25, 26]. Serum levels of amylopsin and lipase did not increase in the study, which suggests that HIFU treatment does not cause pancreatic inflammation.

There are also limitations of the research. Although the safety and efficiency of the technique were encouraging, our results came from retrospective, single institutional, uncontrolled, non-blinded, small study. In the future, we needed larger trials with top-level design not only to further delineate the efficiency of the technique but to delineate rarer adverse events that smaller trials might not have been adequately powered to show.

## Conclusions

In conclusion, our results demonstrated that the combined application of HIFU and radiotherapy might be a promising therapeutic treatment to patients with LAPC. However, the clinical efficacy of this combination therapy needs to be evaluated using double-blind, randomized controlled trials.
